# Chronic Zinc Exposure Decreases the Surface Expression of NR2A-Containing NMDA Receptors in Cultured Hippocampal Neurons

**DOI:** 10.1371/journal.pone.0046012

**Published:** 2012-09-25

**Authors:** Jia Zhu, Chong-Yu Shao, Wei Yang, Xiao-Min Zhang, Zhen-Yong Wu, Liang Zhou, Xin-Xin Wang, Yun-Hong Li, Jun Xia, Jian-Hong Luo, Ying Shen

**Affiliations:** 1 Department of Neurobiology, Key Laboratory of Medical Neurobiology of Ministry of Health, Zhejiang Province Key Laboratory of Neurobiology, Zhejiang University School of Medicine, Hangzhou, People’s Republic of China; 2 Department of Biochemistry, Hong Kong University of Science and Technology, Clear Water Bay, Kowloon, Hong Kong, People’s Republic of China; 3 Department of Neurobiology, Center of Scientific Technology, Cranial Cerebral Disease Lab, Ningxia Medical University, Yinchuan, People’s Republic of China; Oregon Health & Science University, United States of America

## Abstract

**Background:**

Zinc distributes widely in the central nervous system, especially in the hippocampus, amygdala and cortex. The dynamic balance of zinc is critical for neuronal functions. Zinc modulates the activity of N-methyl-D-aspartate receptors (NMDARs) through the direct inhibition and various intracellular signaling pathways. Abnormal NMDAR activities have been implicated in the aetiology of many brain diseases. Sustained zinc accumulation in the extracellular fluid is known to link to pathological conditions. However, the mechanism linking this chronic zinc exposure and NMDAR dysfunction is poorly understood.

**Methodology/Principal Findings:**

We reported that chronic zinc exposure reduced the numbers of NR1 and NR2A clusters in cultured hippocampal pyramidal neurons. Whole-cell and synaptic NR2A-mediated currents also decreased. By contrast, zinc did not affect NR2B, suggesting that chronic zinc exposure specifically influences NR2A-containg NMDARs. Surface biotinylation indicated that zinc exposure attenuated the membrane expression of NR1 and NR2A, which might arise from to the dissociation of the NR2A-PSD-95-Src complex.

**Conclusions:**

Chronic zinc exposure perturbs the interaction of NR2A to PSD-95 and causes the disorder of NMDARs in hippocampal neurons, suggesting a novel action of zinc distinct from its acute effects on NMDAR activity.

## Introduction

The brain has a considerable zinc content with the highest concentration in the hippocampus, amygdala and cortex [1]. In neurons, zinc binds tightly to numerous enzymes, structural proteins and transcription factors [2]. Zinc is also buffered by the metallothioneins [3] and sequestered in mitochondria [4], [5]. In glutamatergic neurons, zinc is present at up to millimolar concentration in presynaptic vesicles and is released from these neurons with glutamate and taken up by presynaptic axon terminals, postsynaptic neurons and neighbouring astrocytes [6]. All these sources of zinc contribute to the dynamic balance of zinc, which is critical for its functions in neurons. Increasing evidence indicates that zinc imbalance plays important roles in the pathophysiologic progress [7], [8]. Marked increase of zinc caused by brain injury induces acute neurotoxicity and cell death [6], [9]. In addition, a phased accumulation of zinc is shown in neurodegenerative diseases, such as Alzheimer’s disease (AD), Parkinson disease (PD) and amyotrophic lateral sclerosis (ALS) [10].

N-methyl-D-aspartate receptor (NMDAR) is the predominant molecule for controlling synaptic plasticity and learning and memory in the central nervous system (CNS) [11]. It is well-known that zinc acutely and allosterically regulates the activity of NMDARs [6], [12], [13]. Zinc suppresses the NR2A-containing NMDARs by either high-affinity (5–300 nM) and voltage-independent inhibition or low-affinity (45–79 µM) and voltage-dependent inhibition [14]–[17]. Differently, zinc inhibits the NR2B-containing NMDARs at the micromolar level and in a voltage-independent manner [14], [15], [18], [19]. Zinc plays important roles in neuronal excitability [20], [21] and synaptic plasticity [22], [23] through the rapid inhibition of NMDARs.

Inappropriate activity of NMDARs is implicated in the aetiology of neurodegenerative diseases [24], [25]. While increased zinc is shown in neurodegenerative diseases [10], it is unknown if this zinc imbalance links to NMDAR dysfunction. The present work was undertaken to remedy this omission. We chronically treated the cultured hippocampal neurons with 100 nM zinc. Results derived from immunostaining and electrophysiology demonstrated that chronic zinc exposure specifically decreased the surface expression of NR2A-containing NMDARs and the currents mediated by them. The mechanism of zinc effects included the disruption of the physical association of the NR2A-PSD95-Src complex. Our data indicated a novel action of zinc distinct from its acute effects on NMDARs.

## Results

### NBQX+nimodipine Protects Hippocampal Neurons in Chronic Zinc Treatment

**Figure 1:**
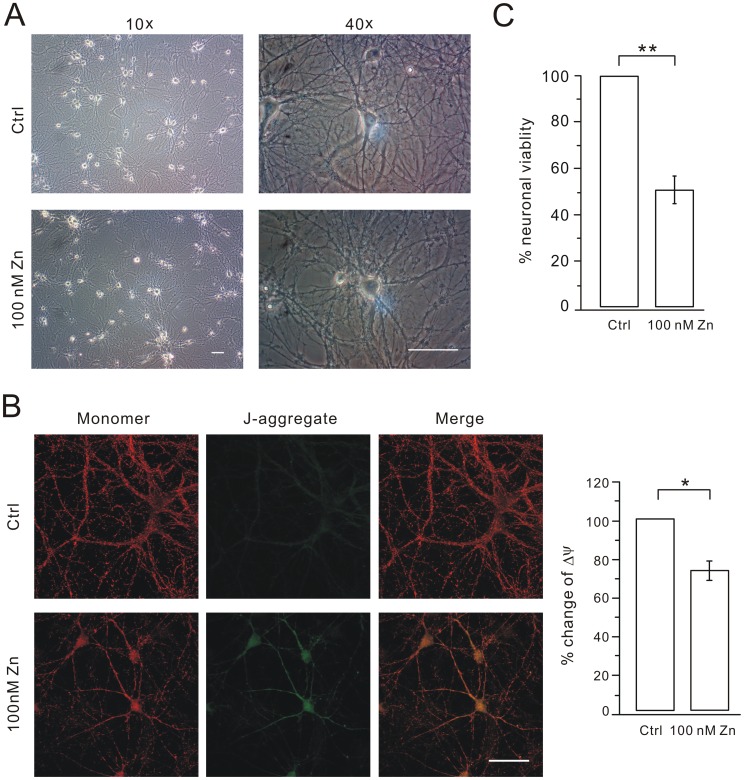
100 nM zinc alone induces cell death. (A) Typical 10× and 40× bright field pictures of hippocampal neurons of control (Ctrl) and 100 nM zinc-treated (100 nM Zn) groups at DIV11. Scale bar, 50 µm. (B) Mitochondrial function was measured in control (Ctrl) and zinc-treated (100 nM Zn) neurons. The red and green signals represent the JC-1 monomer (monomer) and JC-1 J-aggregate (J-aggregate), respectively. Merge is the superimposition of red and green signals. Scale bar, 50 µm. The bar graphs in the right show the mitochondrial membrane potentials (Δψ) of cultured cells in Ctrl and 100 nM Zn group were calculated as the fluorescence ratio of red to green. Y axis represents the % change of Δψ (73.2±4.7%) in 100 nM Zn compared to control. (C) MTT assay derived from cells treated with control (Ctrl) and 100 nM zinc alone at DIV11. The ratio (100 nM zinc; 51±6%; N = 3) was calculated by normalization to control. *, P<0.05.

We first investigated whether hippocampal neurons suffered severe neurotoxicity after the chronic treatment with zinc. Zinc alone was applied at 100 nM in cultured hippocampal neurons from DIV7 to DIV11 and the condition of the cells worsened (Figure 1A). It was easily to identify that, after zinc treatment, the cell bodies became phase-dark, swollen and granular, and cell processes developed a beaded appearance (Figure 1A). We also examined the distribution and function of mitochondria because their morphological transition is an early indicator of cell death [26]. JC-1 forms J-aggregates responding to a low membrane potential. Thus, change of JC-1 color is a quantitative indicator of the transition of mitochondrial function [26]. We found that the fluorescence of JC-1 was red and mitochondria were equally distributed in the soma and dendrites in control cells. Only very weak green fluorescence was observed in these cells (Figure 1B). In zinc-treated cells, there was a significant increase of the green signal, indicating an increase of JC-1 aggregation. Meanwhile, mitochondria were mainly distributed in the soma and main shafts (Figure 1B). There was 26.8% decrease in the averaged Δψ (ratio of red to green) in zinc-treated cells (n = 15) compared to control (n = 15) (P<0.05; Figure 1B). Next, colorimetric 3-(4,5-dimethylthiazol-2yl)-2,5-diphenyltetrazolium bromide (MTT) assay was used to measure neuronal viability when cells were treated with 100 nM zinc. We found that cell viability was enormously reduced in the treatment with zinc (Figure 1C). Taken together, these results suggested that cultured hippocampal neurons suffer severe toxicity when they are treated with zinc alone.

**Figure 2:**
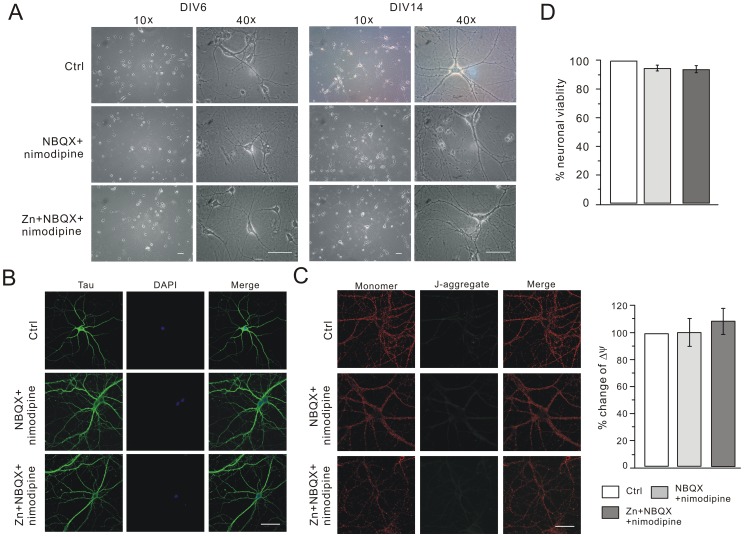
NBQX+nimodipine prevent cell death in chronic zinc exposure. (A) Typical 10× and 40× bright field pictures of hippocampal neurons of control, NBQX+nimodipine and Zn+NBQX+nimodipine groups at DIV6 and DIV 14. Scale bar, 50 µm. (B) Hippocampal cells were immunostained with tau (green) and DAPI (blue) in control, NBQX+nimodipine and Zn+NBQX+nimodipine groups at DIV14. Merge is the superimposition of Tau and DAPI signals. Note that there was no difference in the dendritic branching and nucleus morphology among three groups. Scale bar, 50 µm. (C) Mitochondrial function was measured in control, NBQX+nimodipine and Zn+NBQX+nimodipine groups. The red and green signals represent the JC-1 monomer (monomer) and JC-1 J-aggregate (J-aggregate), respectively. Merge is the superimposition of red and green signals. Scale bar, 50 µm. The quantification is shown in the right. The mitochondrial membrane potential (Δψ) of cultured cells in each group was calculated as the fluorescence ratio of red to green. Y axis represents the percent change of Δψ in NBQX+nimodipine (99.4±9.9%; n = 9) and Zn+NBQX+nimodipine (107.9±9.6%; n = 10) compared to control. (D) MTT assay derived from cells in control, NBQX+nimodipine and Zn+NBQX+nimodipine groups at DIV14. The ratios (NBQX+nimodipine and Zn+NBQX+nimodipine) were calculated by normalization to control (N = 3).

α-amino-3-hydroxy-5-methyl-4-isoxazolepropionic acid receptors (AMPARs) and voltage-gated calcium channels (VGCCs) have markedly great zinc permeability [27], [28]. The inhibition of AMPAR and VGCC afford the neuroprotection against the cell death caused by zinc [29]–[31]. We then investigated whether NBQX+nimodipine protects hippocampal neurons. Several manipulations were performed to clarify this issue. First, there was no change of the cell morphology in NBQX+nimodipine and Zn+NBQX+nimodipine groups compared to control (Figure 2A). Second, the dendritic branching was examined by Tau staining. The patterns of Tau staining were very similar among the three groups (Figure 2B), indicating that the dendritic tree developed well. Meanwhile, cell nuclei appeared normal with DAPI (4′,6-diamidino-2-phenylindole 2 hci) staining (Figure 2B). Third, the number of synapses was examined by immunostaining experiments using the synaptic markers proteins PSD-95 and synaptophysin (Figure S1A and S1C). We found that chronic zinc treatment did not alter the distribution of PSD-95 and synaptophysin (Figure S1B and S1D). These data showed that zinc effects on NMDARs were not due to the reduced number of synapses. Fourth, the distribution and function of mitochondria were examined in three groups. The fluorescence of JC-1 was mainly red with very weak green signals. The labeling patterns were also quite similar among three groups, indicating that mitochondrial function was not affected by zinc in the presence of NBQX+nimodipine (Figure 2C). Finally, we examined the neuronal viability when cells were treated with zinc plus NBQX+nimodipine. We found no statistical difference of neuronal viability in all groups (Figure 2D).

Taken together, these results demonstrated that NBQX and nimodipine prevent the zinc-induced toxicity, consistent with previous reports [32], [33]. Therefore, NBQX and nimodipine were added to cultures to prevent cell loss. In order to accurately evaluate the effects of zinc, 5 µM nimodipine+5 µM NBQX (termed ‘NBQX+nimodipine’) and 100 nM zinc+5 µM NBQX+5 µM nimodipine (termed ‘Zn+NBQX+nimodipine’) were set as the parallel control and experiment groups, respectively. All drugs were added at DIV7 and washed out at DIV14 unless stated otherwise.

### Zinc Exposure Attenuates NR1 Clusters and NMDA Currents

**Figure 3:**
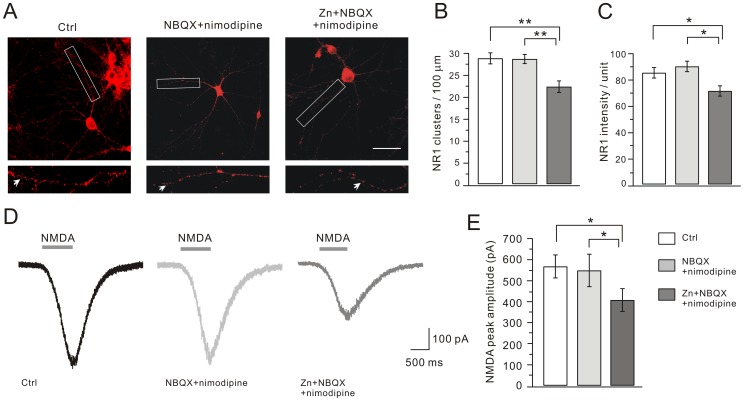
Zinc exposure decreases NR1 clusters and NMDA currents. Cultured hippocampal neurons were immunostained for NR1. (A) Representative images of clustering of NR1 in control, NBQX+nimodipine and Zn+NBQX+nimodipine groups. For each image, a higher magnification shows the dendritic segment (enclosed in white box) studded with numerous clusters, indexed by white arrows. Scale bar, 50 µm. (B) and (C) show the quantification of (A). Both the number and mean intensity of NR1 clusters were significantly decreased after zinc treatment. The mean intensities were 85.4±4.0 (control; n = 27), 90.2±4.0 (NBQX+nimodipine; n = 24), and 71.5±4.0 (Zn+NBQX+nimodipine; n = 27). (D) Representative whole-cell currents in response to agonist solution containing 30 µM NMDA, 10 µM glycine, 1 µM TTX, 10 µM bicuculline, 10 µM NBQX and 1 µM strychnine in cultured pyramidal cells from control, NBQX+nimodipine and Zn+NBQX+nimodipine groups. The averaged peak amplitude of NMDA currents in Zn+NBQX+nimodipine group was reduced, as shown in (E). *, P<0.05, **, P<0.01.

NMDARs are composed of two NR1 and two NR2 subunits in the hippocampus [34], [35]. Therefore, we first detected the immunofluorescent staining of NR1 to measure the distribution of NMDARs. After cells were fixed at DIV14, bright clusters of NR1 staining, typically present on dendritic shafts at the periphery of the dendritic tree, were seen on >90% of the pyramidal neurons (Figure 3A). These clusters were determined by their bright fluorescent intensity that was at least 2-fold brighter than the background dendritic shafts (Figure 3A). The numbers of NR1 clusters were 28.9±1.2 per 100 µm dendrite length in control (n = 30) and 28.7±1.0 in the NBQX+nimodipine group (n = 27) (Figure 3B). Interestingly, there was a 23% decrease of the number of NR1 clusters in the Zn+NBQX+nimodipine group compared to control (22.3±1.1 clusters/100 µm dendrite; n = 28; P<0.01). The mean fluorescent intensity of NR1 clusters was also measured in three groups. Similarly, there was a 22% decrease of the mean fluorescent intensity in the Zn+NBQX+nimodipine group compared to control (Figure 3C). These data indicated that both total NR1 clusters and NR1 subunits contained in each cluster were reduced after the zinc treatment. We also examined the clusters of another membrane receptor, GABA_A_ receptors (GABA_A_Rs) in three groups and found no detectable difference in either the number or mean fluorescent intensity of GABA_A_Rs after zinc treatment (Figure S2).

The immunofluorescence data were not sufficient to judge whether the function of NMDAR was affected. We next measured NMDAR-mediated currents by using whole-cell patch-clamp recordings from cultured neurons. When cells were voltage-clamped at −70 mV, a rapid application of 30 µM NMDA with 10 µM glycine induced inward currents (Figure 3D). On average, the peak currents were similar in the control and NBQX+nimodipine groups (control: 567.7±54.8 pA, n = 38; NBQX+nimodipine: 548.3±77.1 pA, n = 20; Figure 3D). Zinc treatment produced a significant decrease in the peak current with an average amplitude of 407.6±54.4 pA (n = 47; P<0.05 compared to control; Figure 3D), similar to the decrease in the number of NR1 clusters (Figure 3B). Thus, our data showed that chronic zinc treatment attenuated both NR1 clusters and NMDAR currents.

**Figure 4:**
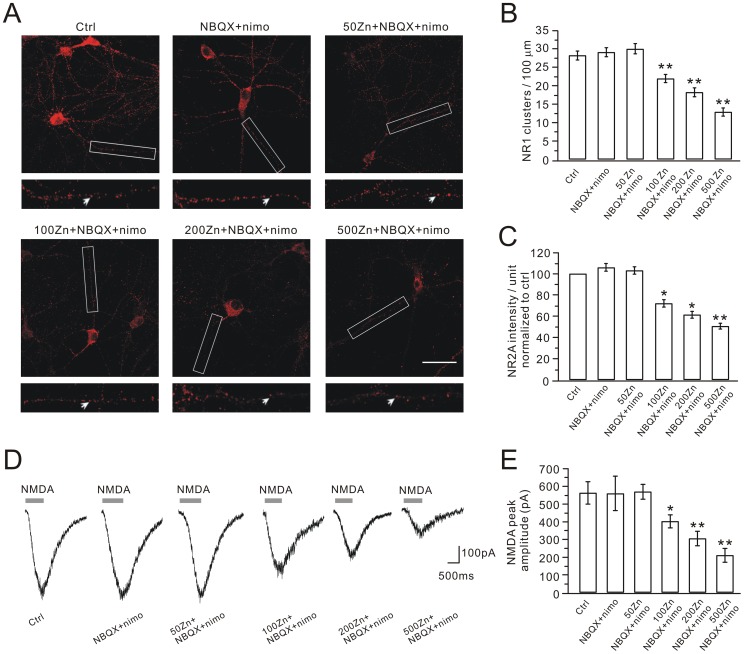
Dose response of zinc effect. (A) Representative images of NR1 clustering in control, NBQX+nimodipine (nimo) and different concentrations (50, 100, 200 and 500 nM) of zinc plus NBQX+nimodipine. For each image, a higher magnification shows the dendritic segment (enclosed in white box) studded with numerous clusters, indexed by white arrows. Scale bar, 50 µm. (B) and (C) show quantifications of NR1 stainings in (A). (D) Representative whole-cell NMDA currents in cultured cells from control, NBQX+nimodipine and different concentrations (50, 100, 200 and 500 nM) of zinc plus NBQX+nimodipine groups. (E) Bar graphs show the average peak amplitudes, which were 563.2±61.3 pA (control, n = 10), 559.6±94.8 pA (NBQX+nimodipine, n = 10), 569.7±40.1 pA (50 nM Zn+NBQX+nimodipine, n = 10), 402.5±35.3 pA (100 nM Zn+NBQX+nimodipine, n = 11), 305.3±39.4 pA (200 nM Zn+NBQX+nimodipine, n = 10) and 210.4±37.4 pA (500 nM Zn+NBQX+nimodipine, n = 10). *, P<0.05; **, P<0.01 compared to control.

To determine the dose-response of zinc effect on NR1 distribution and NMDA currents, we treated hippocampal neurons with a series of concentrations of zinc (50, 100, 200 and 500 nM) with the co-application of NBQX+nimodipine. 50 nM zinc plus NBQX+nimodpine did not change the distribution of NR1 clusters and NMDA currents (Figure 4A and 4B). When the concentration of zinc was greater than 100 nM, Zn+NBQX+nimodipine significantly down-regulated both NR1 clusters and NMDA current (Figure 4B, 4C, 4D and 4E). These results demonstrated that zinc effect on NMDARs was dose-dependent and 100 nM was a critical concentration. Thus, 100 nM was chosen to detect zinc effect on NMDARs in our work.

### Zinc Exposure Down-regulates NR2A-, but not NR2B-containing NMDARs

**Figure 5:**
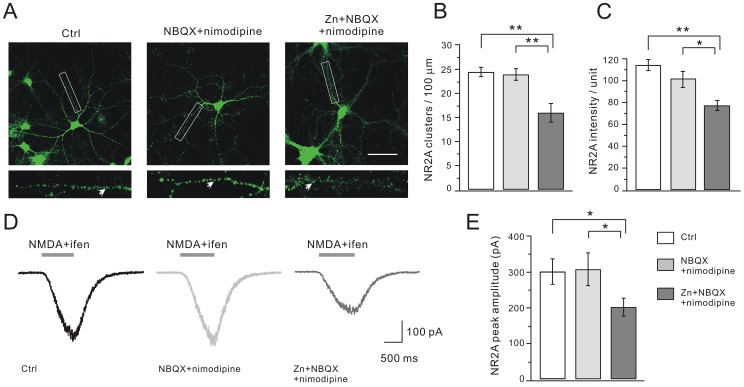
Zinc exposure attenuates NR2A clusters and currents. Neurons were immunostained for NR2A. (A) Representative images of clustering of NR2A in control, NBQX+nimodipine and Zn+NBQX+nimodipine groups. Higher magnification views are shown of the dendritic branches studded with numerous clusters enclosed in white boxes. Scale bar, 50 µm. (B) and (C) show the quantification data of (A). Both the number and mean intensity of NR2A clusters significantly decreased after zinc treatment. The numbers of NR2A clusters were 24.4±1.0 (control, n = 26), 23.9±1.2 (NBQX+nimodipine, n = 24), and 16.0±1.9 (Zn+NBQX+nimodipine, n = 17) per 100 µm dendrite length. The mean intensities were 114.4±5.1 (control; n = 26), 101.9±7.1 (NBQX+nimodipine; n = 24), and 77.4±4.6 (Zn+NBQX+nimodipine; n = 21). (D) Representative NR2A currents in response to co-application of the agonist solution plus ifenprodil. The averaged peak amplitude of NR2A currents in the Zn+NBQX+nimodipine group was significantly reduced, as shown in (E). Averaged peak currents were 302.4±35.3 pA (control, n = 19), 308.2±46.3 pA (NBQX+nimodipine, n = 15) and 202.4±24.1 pA (Zn+NBQX+nimodipine, n = 17). *, P<0.05, **, P<0.01.

NR2A and NR2B subunits are the major NR2 subunits in hippocampal cells [36]. We first determined the effects of chronic zinc treatment on NR2A using immunostaining and electrophysiology. Similar to NR1, bright clusters of NR2A were easily identified on dendritic tree at DIV14 (Figure 5A). Statistics showed that the numbers of NR2A clusters were similar in the control and NBQX+nimodipine groups (Figure 5A). Zinc exposure significantly decreased the number of NR2A clusters (Figure 5B). The mean fluorescent intensity of NR2A clusters was also decreased after zinc exposure (Figure 5C). We then recorded whole-cell NR2A currents by applying 30 µM NMDA with 10 µM glycine and 150 nM ifenprodil, a NR2B antagonist (Ascent Scientific, Bristol, UK) [37], as shown in Figure 5D. On average, the peak currents in the control and NBQX+nimodipine groups were similar (Figure 5E). There was a 33% decrease of the NR2A current in the zinc group (Figure 5E), similar to the decrease of NR2A clusters (Figure 5B).

**Figure 6:**
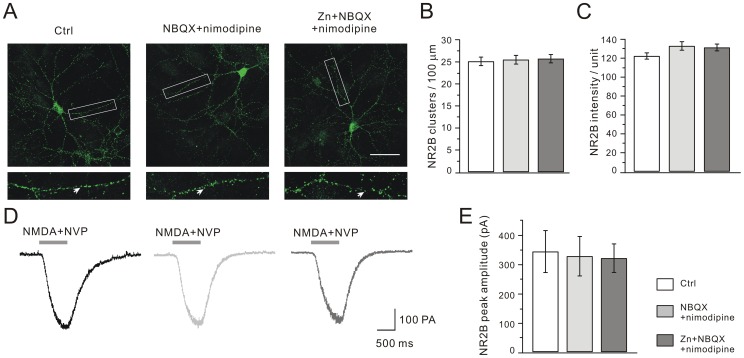
Zinc does not affect NR2B distribution and current. (A) Representative images of clustering of NR2B in control, NBQX+nimodipine and Zn+NBQX+nimodipine groups. Higher magnification views are shown of the dendritic branches studded with numerous clusters enclosed in white boxes. Scale bar, 50 µm. (B) and (C) show the quantification of (A). The numbers of NR2B clusters were 25.1±1.0 (control, n = 37), 25.5±1.0 (NBQX+nimodipine, n = 36), and 25.7±1.0 (Zn+NBQX+nimodipine, n = 37) per 100 µm dendrite length. The mean intensity of NR2B clusters was 122.4±3.3 (control, n = 25), 133.0±4.5 (NBQX+nimodipine, n = 25) and 131.3±3.6 (Zn+NBQX+nimodipine, n = 23). (D) Representative NR2B currents in response to co-application with the agonist plus NVP-AAM077. (E) Statistics of NR2B currents. Averaged peak currents were 342.7±71.7 pA (control, n = 38), 327.0±67.3 pA (NBQX+nimodipine, n = 20) and 320.0±48.5 pA (Zn+NBQX+nimodipine, n = 20).

We next explored the zinc effects on NR2B clustering and currents. It was interesting to find that neither the number nor the mean intensity of NR2B clusters changed after zinc treatment (Figure 6A, 6B and 6C). Likewise, we recorded whole-cell NR2B currents by applying 30 µM NMDA with 10 µM glycine and 40 nM NVP-AAM077 (NR2A antagonist) [38]. Our results showed that NR2B currents were unaltered after zinc exposure (Figure 6D and 6E). These data showed that chronic zinc treatment specifically affected NR2A-containing NMDARs.

### Synaptic NR2A-containing NMDARs are Reduced after Zinc Exposure

**Figure 7:**
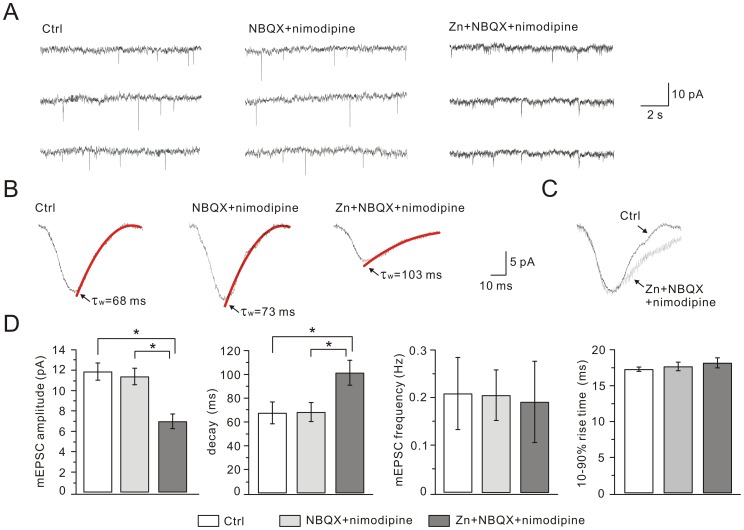
Zinc treatment inhibits NMDA mEPSCs. (A) Representative NMDA mEPSCs recorded from control, NBQX+nimodipine and Zn+NBQX+nimodipine groups in extracellular medium containing TTX, bicuculline, and NBQX in the absence of Mg^2+^. (B) Averaged NMDA mEPSCs with superimposed double exponential fitting of the decay phase and indication of the resulting weighted time constant (τ_w_, red traces). (C) NMDA mEPSCs of control shown in black and Zn+NBQX+nimodipine in grey. For comparison, grey trace was normalized to black trace according to the peak. (D) Summaries of peak amplitude, frequency, τ_w_ and 10–90% rise-time of NMDA mEPSCs recorded from control, NBQX+nimodipine and Zn+NBQX+nimodipine groups. The averaged amplitudes of NMDA mEPSCs were 11.9±0.8 pA (control), 11.4±0.8 pA (NBQX+nimodipine) and 7.0±0.7 pA (Zn+NBQX+nimodipine). The averaged frequencies were 0.21±0.08 Hz (control), 0.21±0.05 Hz (NBQX+nimodipine) and 0.19±0.09 Hz (Zn+NBQX+nimodipine). The values of τ_w_ were 67.7±9.2 ms (control), 68.4±8.0 ms (NBQX+nimodipine) and 101.5±10.5 ms (Zn+NBQX+nimodipine). The average 10–90% rise-times were 17.3±0.3 ms (control), 17.7±0.6 ms (NBQX+nimodipine) and 18.2±0.7 ms (Zn+NBQX+nimodipine). 13 cells were used for control, 12 for NBQX+nimodipine and 12 for Zn+NBQX+nimodipine. *, P<0.05.

Synaptic NMDARs are critical for the induction of synaptic plasticity in hippocampal neurons [36]. To investigate whether synaptic NMDARs are affected by zinc exposure, we recorded NMDAR-mediated miniature EPSCs (mEPSCs) in three groups at DIV14. At a command voltage of −70 mV, NMDA mEPSCs were recorded for 10–20 min in an external solution with 1 µM TTX, 10 µM bicuculline, 10 µM NBQX, 1 µM strychnine and without Mg^2+^. Typical traces from three individual cells were shown in Figure 7A. We found that the amplitude decreased, but the frequency and the 10–90% rise-time of the mEPSCs remained unchanged in Zn+NBQX+nimodipine cells (Figure 7D). These data indicated that postsynaptic NMDARs were reduced after zinc exposure. Measurement of the weighted time constant of decay (τ_w_) revealed that the decay became slower in Zn+NBQX+nimodipine cells (Figure 7B and 7C). Since the decay time of NR2B currents is slower than that of NR2A currents [35], this result implied that chronic zinc exposure specifically decreased synaptic NR2A-containing NMDARs while NR2B-containing NMDARs were less affected. Addition of the selective NMDAR antagonist, DL-AP5 (200 µM) completely abolished NMDA mEPSCs (n = 8, data not shown).

### Zinc Exposure Decreases the Surface Expression of NR1 and NR2A

**Figure 8:**
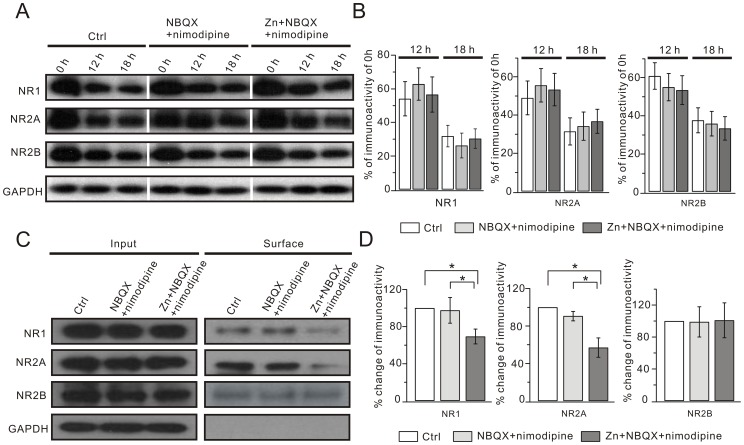
Zinc reduces surface expression of NR1 and NR2A. (A) Hippocampal neurons derived from control, NBQX+nimodipine and Zn+NBQX+nimodipine groups were incubated in 35 µg/ml CHX. Total expressions of NR1, NR2A and NR2B were measured at 0 hr, 12 hr or 18 hr in the CHX treatment. (B) shows the quantification of (A). (C) Surface NR1, NR2A and NR2B derived from cultured hippocampal neurons were isolated by biotinylation assay and detected by anti-NR1, anti-NR2A and anti-NR2B antibodies. Surface NR1 and NR2A, but not NR2B, were notably lower in the Zn+NBQX+nimodipine group, while total NR1, NR2A and NR2B did not change. (D) Quantifications of (C). Percentage changes of surface signal intensities versus control were: NR1, 97.6±13.8 (NBQX+nimodipine) and 69.4±8.0 (Zn+NBQX+nimodipine); NR2A, 87.7±4.9 (NBQX+nimodipine) and 57.0±14.3 (Zn+NBQX+nimodipine); and NR2B, 99.0±19.0 (NBQX+nimodipine) and 101.0±21.8 (Zn+NBQX+nimodipine). All experiments were performed at least 3 times (N > = 3). *, P<0.05.

The reduced NMDAR currents after zinc chronic treatment might be due to the decrease of either NMDAR subunit protein synthesis or its surface expression. In order to determine which mechanism was responsible for zinc effects, we evaluated the synthesis of NR1, NR2A and NR2B. Cycloheximide (CHX) is known to effectively inhibit protein translation and has been used to investigate the protein degradation [39]. Therefore, we compared NR1, NR2A and NR2B expression in the absence of CHX. Our data showed that the total expression of the three subunits was not changed by zinc exposure (Figure 8A). We then treated the three groups with 35 µg/ml CHX. The expressions of NR1, NR2A and NR2B were reduced at 12 h and 18 h of CHX treatment, but the rates of decrease were constant among the three groups (Figure 8A). Because both the total expression and the rate of decrease of NR1, NR2A and NR2B were not changed (Figure 8B), we concluded that the synthesis efficacy and half-life of the three subunits are not changed by zinc exposure.

We next investigated the surface expression of the three subunits in hippocampal neurons. Interestingly, our results showed that the amounts of both NR1 and NR2A on the cell surface significantly decreased in Zn+NBQX+nimodipine neurons, while NR2B surface expression was not affected. The decreases of surface NR1 and NR2A in the Zn+NBQX+nimodipine group were 31% and 43%, respectively, compared to control (Figure 8C and 8D). Thus, zinc exposure attenuated NR1 and NR2A, but not NR2B, on the surface of hippocampal neurons, implying that the down-regulation of NR2A-containing NMDARs might be due to their decreased surface expression.

### Zinc Exposure Disrupts NR2A-PSD-95-Src Interaction and Causes Down-regulation of NR2A-contaning NMDARs

**Figure 9:**
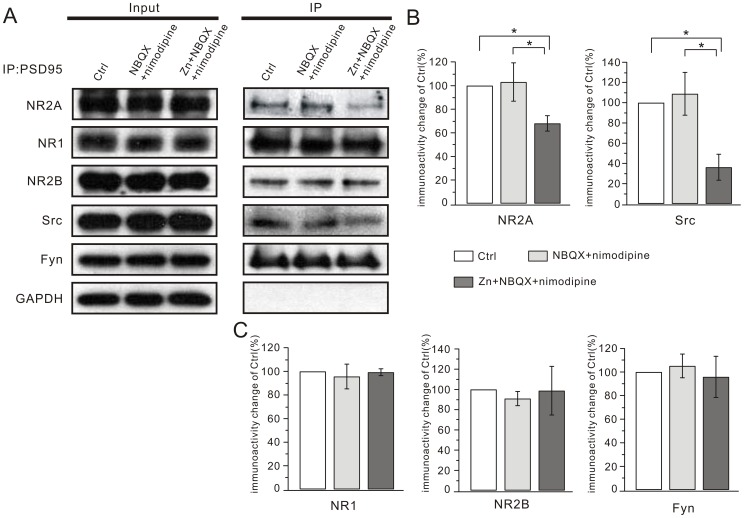
Zinc disrupts the NR2A-PSD-95-Src complex. (A) Lysates were immunoprecipitated with anti-PSD-95 antibody in control, NBQX+nimodipine and Zn+NBQX+nimodipine groups. The immunoprecipitates were probed with the anti-NR2A (NR2A), anti-NR1 (NR1), anti-NR2B (NR2B), anti-Src (Src) and anti-Fyn (Fyn) antibodies. The Input lane was loaded with total protein (20 µg) derived from cultured hippocampal neurons. (B) Supernatant samples were immunoprecipitated with anti-PSD-95 antibody in different groups, which showed the clearance of PSD95 after Co-IP experiment. All experiments were performed at least 3 times (N > = 3) and the summarized data are shown in (C) and (D). Y axes in (C) and (D) represent the percentage change of each co-precipitated protein (labeled on X axes) relative to the corresponding control. *, P<0.05.

The reduced currents and surface expression of NR2A suggested that NR2A might be unstable on the cell surface after zinc treatment. It is known that NMDAR subunits have a long cytoplasmic C-terminal domain that associates with scaffolding proteins, adaptor proteins and signaling enzymes, which together constitute the NMDAR complex [40], [41]. Extracellular and intracellular signaling molecules directly or indirectly regulate NMDARs through this complex [41]. PSD-95, a major component of the NMDAR complex, has a high binding affinity to NR2A and increases the expression of synaptic NR2A by anchoring the receptors into a molecular scaffold [41], [42]. To explore whether zinc exposure modulates the interaction between PSD-95 and NR2A, we performed co-immunoprecipitation (Co-IP) to assess the amount of NR2A precipitated by PSD-95. We found that the interaction between PSD-95 and NR2A was dramatically reduced after zinc exposure (change of immunoactivity: 68.0±6.6%; N = 6; P<0.05 compared to control; Figure 9A and 9B). Interestingly, we found that zinc did not influence the interaction of PSD-95 with NR1 or NR2B (Figure 9A and 9C).

Protein tyrosine kinase Src, another component of the NMDAR complex, is an important regulator of NR2A [41], [43]–[45]. Src binds with PSD-95 in the postsynaptic density [46], [47]. We performed Co-IP to explore the effect of zinc on the interaction between PSD-95 and Src, and found that Src precipitated by PSD-95 decreased after zinc exposure (change of immunoactivity: 36.2±12.9%; N = 7; P<0.05 compared to control; Figure 9A and 9B). However, another Src family kinase Fyn, which has a high affinity for NR2B [47], did not show decreased precipitation after zinc treatment (Figure 9C). PSD-95 and Src participate in the NMDAR complex and play important roles in regulating NMDAR function. Based on the results, we concluded that the down-regulation of NR2A-containing NMDARs might be induced by the dissociation of NR2A-PSD-95-Src caused by chronic zinc exposure.

## Discussion

In the present work, we found that chronic exposure to 100 nM zinc with NBQX and nimodipine reduced the numbers of NR1 and NR2A clusters and inhibited NR2A-mediated currents. In contrast, zinc did not affect the expression of NR2B and GABA_A_R. These results suggested that chronic zinc exposure specifically influenced NR2A-containing NMDARs. We also noticed that this effect of zinc is dependent on its concentration, because our data showed that lower dose of zinc (<100 nM; Figure 4) did not induce any change on NMDARs. Surface biotinylation assay showed that zinc exposure attenuated NR1 and NR2A on the plasma membrane. Moreover, Co-IP experiments showed that zinc reduced the interactions of PSD-95 with NR2A and Src. NR2A-PSD-95-Src complex is essential for the stability of NMDARs on the plasma membrane [35], [41]. Therefore, we concluded that chronic zinc exposure dissociated the NR2A-PSD-95-Src complex and caused the decrease of membrane NR2A-containg NMDARs. Our work suggested a novel action of chronic zinc exposure on NMDARs, distinct from its acute allosteric regulations [6], [15], [16], [48]–[50].

In CNS, zinc is significantly uptaken by neighboring astrocytes *in vivo* so that the extracellular zinc level is kept at normal [51], [52]. Although the neuronal culture was an efficient model for our study, a disadvantage was that neurons were more susceptive to neurotoxicity because of lack of glial uptaken system. It is reported that NBQX and nimodipine prevent cell death in long-term neuronal culture [32], [33]. Therefore, NBQX+nimodipine were supplemented in cultures so that zinc effects on NMDARs were able to be observed. Indeed, we showed that cultured cells suffered severe cell death when treated with zinc alone (Figure 1). Applying NBQX and nimodipine decreased the neurotoxicity and cell death caused by zinc (Figure 2). Importantly, the application of NBQX+nimodipine allowed us to assess the effect of zinc on NMDARs, which might be obscured by the neurotoxic effects. In the present work, the effects of zinc might result from its minor influx through NMDARs [53], zinc transporters [54] and exchangers [27], [28]. Unfortunately, we were unable to directly measure the concentration of intracellular zinc because currently there is no efficient method to detect the nanomolar-level zinc inside neurons.

PSD-95 physically associates with NMDARs [55], [56] and plays important roles in NMDAR immobilization, clustering and targeting [57], [58], which is mediated by Src family kinases [59]. PSD-95 promotes the Src tyrosine phosphorylation of NR2A [60], [61]. Thus, Src and PSD-95 work together to play a key role in modulating the stability of NR2A-containing NMDARs on surface [41], [62]. Our work suggested that zinc impaired the stability of NR2A-containing NMDARs on the plasma membrane through the dissociation of NR2A-PSD-95-Src complex. It is unknown how zinc regulates the NR2A-PSD-95-Src complex, but possibly it is mediated by an indirect interaction between zinc and Src. A variety of intracellular signaling molecules have been reported to be involved in the modulation of the NR2A-PSD-95-Src complex [41]. It is noteworthy that some of these signaling molecules are regulated by zinc [63]. For example, it is reported that zinc inhibits receptor protein tyrosinephosphatase α (R-PTP-α) [64], [65], which is required for the dephosphorylation of Src [66]. Nevertheless, an extracellular action of zinc cannot be excluded.

A reversible memory loss is found in the Tg2576 mice, an early stage AD model, in which no neuronal loss or structural damage happens [67]. However, it is unknown how this reversible memory loss takes place. Since a progressive zinc imbalance occurs in neurodegenerative diseases [68]–[70], it is possible, according to current work, that zinc imbalance may reduce membrane expression of NMDARs and result in the loss of synaptic plasticity and memory loss. It will be intriguing to investigate the membrane expression of NMDARs and the effects of zinc chelator in the Tg2576 mice. NR2A-containing NMDARs are required for the LTP induction in hippocampus [36]. Moreover, PSD95 is critical to the trafficking and anchoring of NMDARs [71]. Therefore, NR2A-PSD95-Src complex plays important roles in LTP induction. One caveat in the present work is that we did not examine the effect of chronic zinc exposure on long-term plasticity (LTP) because it was hard to apply LTP conditioning protocol in the context of zinc exposure (data not shown). However, it can be deduced that chronic zinc treatment might hurt the induction of LTP.

On the other hand, a small elevation of zinc triggers anti-apoptotic processes in hippocampal cells [72], implying that accumulated zinc can be neuroprotective. It is shown that the NR2A-PSD-95-Src complex plays critical roles in ischemic injury [61], [73]. Inhibition of interactions among NR2A and Src with PSD-95 prevents cell death after transient ischemia [74]. Therefore, sustained zinc exposure may influence the neuronal integrity by decreasing functional NMDARs in sub-lethal conditions.

## Materials and Methods

### Hippocampal Culture and Pharmacological Treatments

All animal procedures were approved by the Animal Experimentation Ethics Committee of Zhejiang University. All chemicals were from Sigma (St. Louis, MO) unless stated otherwise. Hippocampal neuronal cultures were prepared according to the protocols from a previous study [75]. Hippocampi were dissected from embryonic day 17–18 Sprague-Dawley rats and dissociated neurons were plated on glass coverslips coated with 100 µg/ml poly-D-lysine at densities of 2000–3000 cells/cm^2^ (electrophysiology and immunostaining experiments) or 7000–10000 cells/cm^2^ (biochemical experiments). Neurons were cultured in Neurobasal medium supplemented with 2% B-27, 2 mM L-alanyl-glutamine and 2.5 µM AraC (Invitrogen, Carlsbad, CA). The culture medium was renewed every 3–4 days. All cultures were maintained at 37°C in a humidified incubator gassed with 95% O_2_ and 5% CO_2_. Cells were viewed using an inverted phase contrast microscope (Olympus, Tokyo, Japan), and images were digitally captured using a CCD RT digital camera and compiled using Adobe Photoshop 6.0 software.

### Neuronal Viability Assay

MTT assay was used to assess the percentage of viable cells after pharmacological treatments, as previously described [76]. After treatments, culture medium in the 96-well plates was replaced by 100 µl fresh MTT solution (0.5 mg/ml) dissolved in Neurobasal medium. After 3 h incubation at 37°C, the supernatant was removed, the formazan salt crystals were dissolved in 150 µl dimethyl sulfoxide (DMSO). The viability of neurons was measured by the absorbance at 570 nm. The neuronal survival ratio was calculated by normalization to control.

### Measurement of Mitochondrial Membrane Potential

The fluorescent, lipophilic and cationic probe, JC-1 (Beyotime, Shanghai, China), was used to measure the mitochondrial membrane potential (Δψ) [26], [77]. Cells were incubated with JC-1 (5 µg/ml) for 20 min at 37°C and then rinsed twice with the JC-1 staining buffer. Images were viewed and scanned using a Fluoview FV1000 Confocal microscope (Olympus) at 488 nm excitation and 530 nm emission for green, and at 543 nm excitation and 590 nm emission for red. The Δψ was calculated as the fluorescence ratio of red (JC-1 monomer) to green (JC-1 J-aggregate).

### Immunocytochemistry and Quantitation

For immunostaining, neurons were fixed in pre-cooled (−20°C) methanol for 5 min. Cells were pre-incubated in 10% bovine serum albumin and 0.4% Triton-X-100 in PBS for 1 h at room temperature and immediately exposed to the primary antibodies overnight at 4°C. The primary antibodies used for immunostaining were anti-NR1 (1∶500), anti-NR2A (1∶50), anti-NR2B (1∶250), anti-GABA_A_ (1∶300), anti-Tau (1∶500), anti-PSD95 (1∶1000), and anti-synaptophysin (1∶500). Then the cultures were rinsed in PBS and incubated with fluorochrome-conjugated secondary antibodies (Alex543, 1∶1000; Alex488, 1∶1000; Invitrogen) for 1 h at room temperature. Immunocytochemistry images were obtained with a Confocal microscope (Olympus) at a resolution of 1024×1024 pixels. All the parameters used in Confocal microscopy were consistent in each experiment, including the laser excitation power, detector and off-set gains and the pinhole diameter. MetaMorph 5.0 software (Universal Imaging, West Chester, PA) was used to analyze cell images. We chose the first-order, second-order and third-order dendritic shafts in this measurement. The mean fluorescent intensity of the target dendritic shaft was calculated and set as the background. Usually, the variation of mean intensities did not exceed 10% (data not shown). The 2-fold value of the mean intensity of the dendritic shaft was set as the threshold for detecting clusters. The imaging data analysts were blind to the experimental conditions until the data were integrated.

### Co-immunoprecipitation

Cells were lysed in ice-cold 10 mM Tris-HCl buffer, pH7.4. Lysates were solubilized by the addition of 0.10 volume of 10% sodium deoxycholate in 500 mM Tris-HCl, pH 9.0, and incubated at 37°C for 30 min. A 0.1 volume of 1% Triton-X-100/50 mM Tris-HCl (pH 9.0) was added and the preparations were dialyzed against binding buffer (50 mM Tris-HCl, pH 7.4, 0.1% Triton-X-100) overnight at 4°C. The protein concentration in the supernatant was determined using BCA kit assay (Thermo, Rockford, IL) after centrifugation at 37,000×g at 4° for 30 min. Decimus supernatant was used for input and others for immunoprecipitation. Anti-PSD-95 antibody was incubated with protein A-sepharose (GE Healthcare, Waukesha, WI) at 10–20 µg antibody/10 µl sepharose beads for 2 h in 50 mM Tris-HCl. IgG-mouse (Invitrogen) was used as the negative control. The beads were washed with a binding buffer and stored at 4°C. The solubilized preparations were incubated with anti-PSD-95 antibody pre-coupled to protein A-sepharose beads. The proteins on the beads were extracted with 2× sodium dodecyl sulfate (SDS) sample buffer. All samples were boiled for 5 min before Western blot assay.

### Surface Biotinylation

Cell cultures were rinsed twice with PBS containing 1 mM MgCl_2_ and 0.5 mM CaCl_2_ (pH adjusted to 7.4), then incubated with 1 mg/ml sulfo-NHS-LC-biotin (Thermo) for 30 min at 4°C. Cells were quenched with 100 µM glycine for 15 min at 4°C. For avidin pull-down, cells were lysed in ice-cold RIPA buffer (0.1% SDS, 0.5% sodium deoxycholate, 1% Triton-X-100) and centrifuged for 20 min at 4°C. The concentration of supernatant was measured using BCA assay and the proteins were incubated with NeutrAvidin beads (Thermo) for 2 h at 4°C. The beads were washed twice with ice-cold lysis buffer. Biotinylated proteins were extracted using 2× SDS sample buffer supplemented with 50 mM dithiothreitol (DTT) for 30 min at 50°C prior to Western blot.

### Western Blot

Equal quantities of protein were loaded and ran on SDS/polyacrylamide gels and transferred to a PVDF membrane (Millipore, Billerica, MA). Blots were blocked with 5% dried milk and then incubated in primary antibody in TBST overnight at 4°C. After being rinsed in milk–TBST, blots were incubated in the horseradish peroxidase-conjugated secondary antibodies (GE Healthcare), reacted with chemiluminescent substrate (Pierce, Rockford, IL), and exposed to film (Kodak, Rochester, NY). The film were digitally scanned, and the signals on the digital images were quantitated using Image-J 1.42q (NIH, Bethesda, MD), as previously reported [78]. GAPDH was set as the internal control.

### Electrophysiology

Whole-cell patch-clamp recordings were performed in cultured hippocampal neurons at room temperature as in previous studies [75], [79]. Cultures were bathed in Mg^2+^-free Ringer’s containing (in mM): 145 NaCl, 3 KCl, 2 CaCl_2_, 10 HEPES, and 10 glucose (310 Osm, pH 7.4 adjusted with NaOH). Patch pipettes were pulled from borosilicate glass with a perpendicular pipette puller (Narishige Instruments, Tokyo, Japan). Pipette series resistance was 3–7 MΩ when filled with an intracellular solution containing (in mM): 135 Cs methanesulfonate, 10 CsCl, 0.2 EGTA, 10 HEPES, 4 Na-ATP, and 0.4 Na-GTP (310 Osm, pH adjusted to 7.3 with KOH). Cells were voltage-clamped at −70 mV using a 700A amplifier (Molecular Devices, Foster City, CA). The analog signals were sampled at 10 kHz and low-pass filtered at 2 kHz using pClamp 9.0 (Molecular Devices). Cells were excluded from the study if series resistance or input resistance varied by more than 15% over the course of an experiment. To measure whole-cell NMDA currents, a rapid solution changer (RSC-160, Biological Science Instruments, Claix, France) was used to apply drug solutions to the recorded cells. The duration of passage from one injector to an adjacent one was set at 10 ms. Solutions in the tubes were fed into the perfusion bath by gravity at a flow rate of 0.5 ml/min. Solutions were applied through a linear array of 9 glass injectors (I.D. 300 µm), positioned at 150–200 µm from the recorded cell. The protocol of drug application was controlled by the analog output from the pClamp program.

### Miniature EPSC

For mEPSC recordings, the analog signals were sampled at 10 kHz. Cells were excluded from the study if series resistance or input resistance varied by more than 15% over the course of an experiment. mEPSCs were recorded in the whole-cell configuration in the presence of 1 µM tetrodotoxin (TTX), 10 µM bicuculline, and 10 µM NBQX. mEPSC offline analysis was conducted using a sliding template algorithm (ClampFit 10, Molecular Devices) [80]. Overlapping events were rejected. Fitting of the decay phase of mEPSCs was performed according to the previous report [81], using a simplex algorithm for least squares exponential fitting routines. Decay times of averaged mEPSCs were fitted with the double exponential equation:
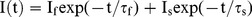
(1)where If and Is are the amplitudes of the fast and slow decay components, and τf and τs are their respective decay time constants. A weighted mean decay time constant was used for the comparisons among groups:
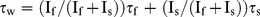
(2)


### Antibodies

Mouse monoclonal PSD95 antibody, rabbit polyclonal Tau, Fyn and synaptophysin antibodies were from Millipore. Mouse monoclonal NR1 antibody was from BD Pharmingen (San Jose, CA). Rabbit polyclonal NR2B antibody was from Alomone Labs (Jerusalem, Israel). Anti-GABA_A_R antibody was from Sigma. Rabbit polyclonal Src antibody was from Cell Signaling (Danvers, MA). Anti-GAPDH antibody was from Abcam (Cambridge, UK). The rabbit polyclonal NR2A antibody was a gift from Dr. Richard Huganir (Johns Hopkins University, Baltimore, MD).

### Statistical Analysis

Statistical differences were determined using one-way ANOVA test. Statistical significance was accepted at P<0.05. Data in the text and figures are presented as mean±SEM. N represents the number of experiments using multiple cultures. n represents the number of tested cells where individual neurons were used.

## Supporting Information

Figure S1 **Zinc does not affect synaptic number.** (A) Representative images of clustering of PSD-95 and synaptophysin in control, NBQX+nimodipine and Zn+NBQX+nimodipine groups. Higher magnification views show the dendritic branches studded with numerous clusters enclosed in white boxes. Scale bar, 50 µm. (B) and (C) show the quantification of the numbers of PSD-95 and synaptophysin clusters. There was no difference of the numbers of both clusters among three groups. PSD-95 clusters per 100 µm dendrite were 96.5±2.3 (Ctrl, n = 39), 95.1±3.7 (NBQX+nimodipine, n = 29) and 101.0±3.2 (Zn+NBQX+nimodipine, n = 28). Synaptophysin clusters per 100 µm dendrite were 91.1±2.9 (Ctrl, n = 39), 92.5±2.7 (NBQX+nimodipine, n = 29) and 92.0±4.5 (Zn+NBQX+nimodipine, n = 28).
(TIF)

Figure S2 **Zinc exposure does not affect GABA_A_R clustering.** (A) Representative images of clustering of GABA_A_R in control, NBQX+nimodipine and Zn+NBQX+nimodipine groups. Higher magnification views show the dendritic branches studded with numerous clusters enclosed in white boxes. Scale bar, 50 µm. (B) and (C) show the quantification of GABA_A_R clustering. Neither the number nor the averaged intensity of GABA_A_R clusters changed after zinc treatment. The numbers of GABA_A_R clusters were 42.1±1.2 (Ctrl, n = 39), 41.9±1.8 (NBQX+nimodipine, n = 45) and 41.9±1.9 (Zn+NBQX+nimodipine, n = 35) per 100 µm dendrite at DIV14. The mean fluorescent intensity of GABA_A_R clusters were 149.2±8.4 (Ctrl, n = 28), 148.3±7.8 (NBQX+nimodipine, n = 30) and 142.2±10.5 (Zn+NBQX+nimodipine, n = 20).
(TIF)
