# Supplementary figures and images for: Chronic Zinc Exposure Decreases the Surface Expression of NR2A-Containing NMDA Receptors in Cultured Hippocampal Neurons
Source: PLoS One. 2012 Sep 25;7(9):e46012. doi: 10.1371/journal.pone.0046012 (PMC3457937; doi:10.1371/journal.pone.0046012)

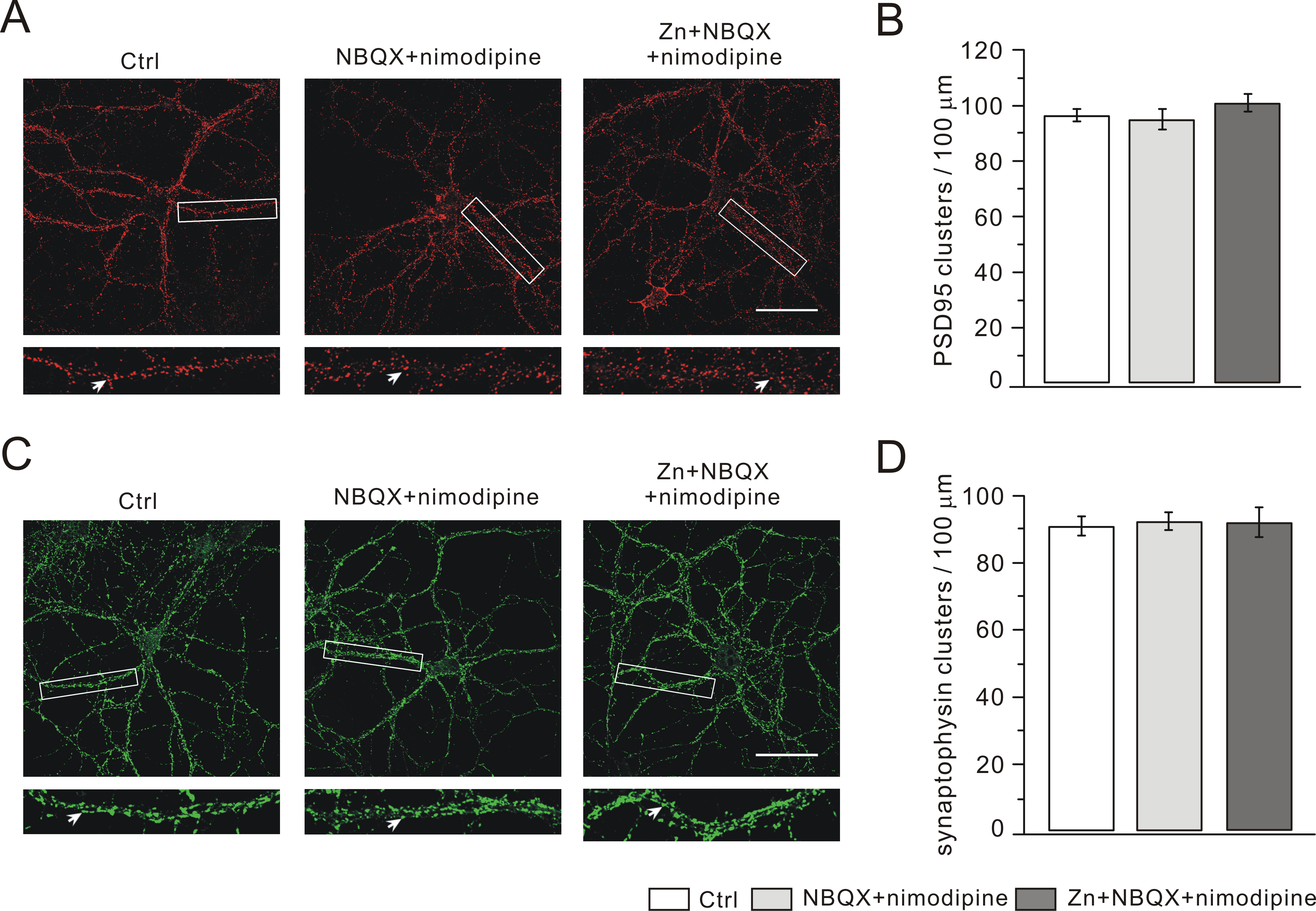

Supplement: Figure S1 — Zinc does not affect synaptic number. (A) Representative images of clustering of PSD-95 and synaptophysin in control, NBQX+nimodipine and Zn+NBQX+nimodipine groups. Higher magnification views show the dendritic branches studded with numerous clusters enclosed in white boxes. Scale bar, 50 µm. (B) and (C) show the quantification of the numbers of PSD-95 and synaptophysin clusters. There was no difference of the numbers of both clusters among three groups. PSD-95 clusters per 100 µm dendrite were 96.5±2.3 (Ctrl, n = 39), 95.1±3.7 (NBQX+nimodipine, n = 29) and 101.0±3.2 (Zn+NBQX+nimodipine, n = 28). Synaptophysin clusters per 100 µm dendrite were 91.1±2.9 (Ctrl, n = 39), 92.5±2.7 (NBQX+nimodipine, n = 29) and 92.0±4.5 (Zn+NBQX+nimodipine, n = 28). (TIF) [file pone.0046012.s001.tif]

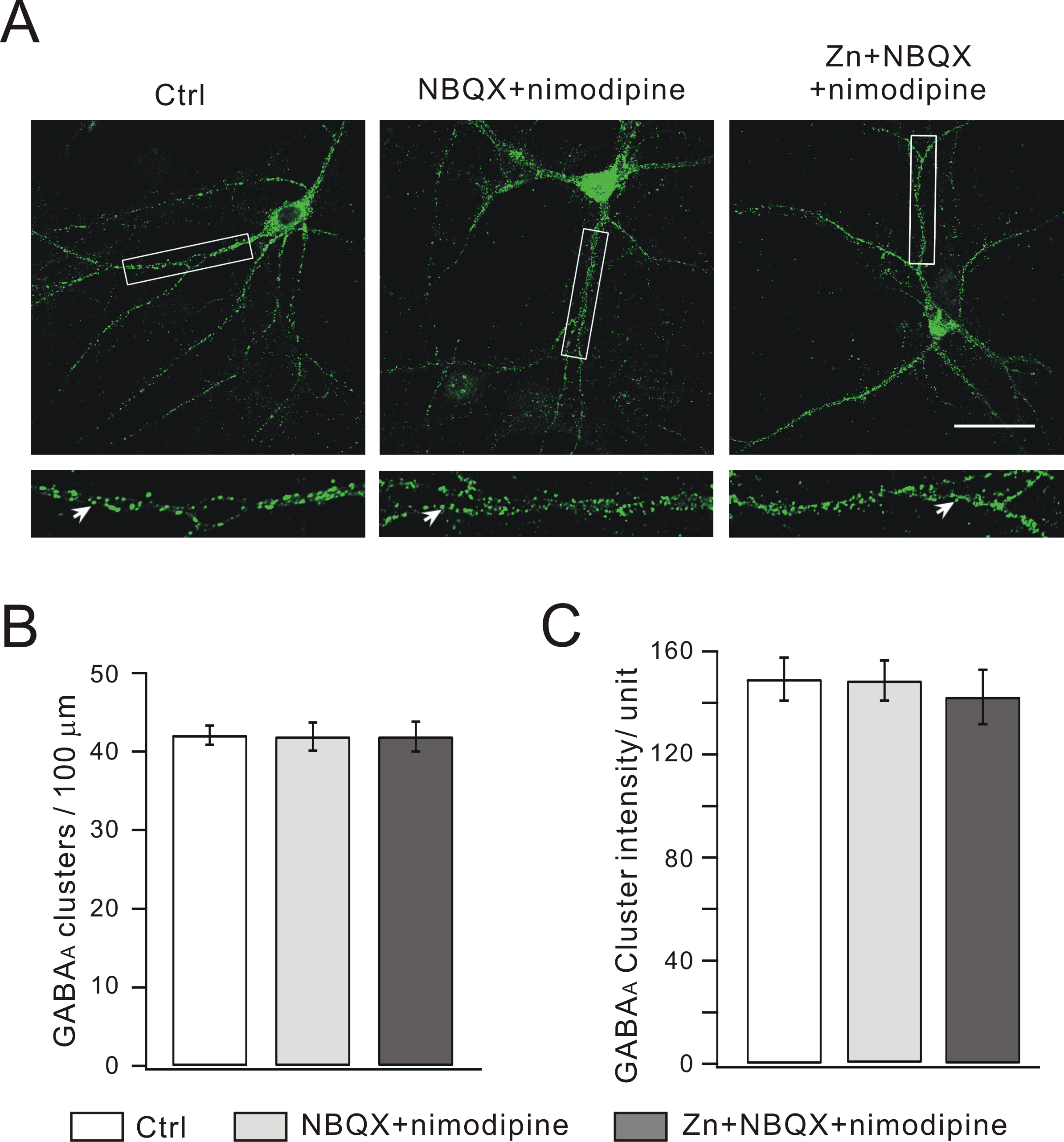

Supplement: Figure S2 — Zinc exposure does not affect GABAAR clustering. (A) Representative images of clustering of GABAAR in control, NBQX+nimodipine and Zn+NBQX+nimodipine groups. Higher magnification views show the dendritic branches studded with numerous clusters enclosed in white boxes. Scale bar, 50 µm. (B) and (C) show the quantification of GABAAR clustering. Neither the number nor the averaged intensity of GABAAR clusters changed after zinc treatment. The numbers of GABAAR clusters were 42.1±1.2 (Ctrl, n = 39), 41.9±1.8 (NBQX+nimodipine, n = 45) and 41.9±1.9 (Zn+NBQX+nimodipine, n = 35) per 100 µm dendrite at DIV14. The mean fluorescent intensity of GABAAR clusters were 149.2±8.4 (Ctrl, n = 28), 148.3±7.8 (NBQX+nimodipine, n = 30) and 142.2±10.5 (Zn+NBQX+nimodipine, n = 20). (TIF) [file pone.0046012.s002.tif]
